# Book Reviews

**Published:** 1894-08

**Authors:** 


### *BOOK REVIEWS.*

---

An Illustrated Dictionary of Medicine, Biology, and Allied Sciences. Including the Pronunciation, Accentuation, Derivation, and Definition of the Terms Used in Medicine and the Allied Sciences. By George M. Gould, A.M., M.D., Author of "The Student's Medical Dictionary"; "12,000 Medical Words Pronounced and Defined;" Editor of the *Medical News;* President, 1893–94, American Academy of Medicine. P. Blakiston, Son & Co., 1012 Walnut street, Philadelphia.

It does look like doctors will not be allowed to lack for dictionaries. Several have been offered us within the last few years, of differing grades of merit.

The one before us is the latest, and in many respects the best that we have seen. This dictionary seeks (1) to include the many thousands of new words and terms that have been introduced into medicine and biology within late years, (2) to encourage a more consistent and phonetic spelling, (3) to give the best pronunciation of words by the simplest method, and (4) within the limits of the volume to give it as nearly an encyclopedic character as possible, not only by supplying the usual pronunciation, derivation and definition of words, but also by showing their logical relations, their bearings, and their practieal importance. These purposes have been well carried out. The spelling as here advocated is certainly a step toward a better system of orthography. In the author's attempt to elaborate words in all of their relations we think he goes further than necessary in some instances. The volume is well illustrated, but is not a picture book. The anatomical cuts are excellent, better than many found in our works on anatomy. But students and doctors will not use their dictionary for an anatomical text-book, so we question the wisdom of going so fully into illustrated anatomy. One valuable feature of the work is the number of useful tables, almost one hundred in all. One table of the various forms of bacteria comprises thirty pages; another table is devoted to the parasites, and is very complete; another is a table of tests, giving the name of the test, reagents employed, reaction, uses, etc.; one of the best is the table on stains, giving the technique of staining, imbedding, mounting, etc. There is also a useful eponymic table of operations and methods employed in surgery, and under the head of "Treatment" there is a similar table.

We have gone over this dictionary somewhat carefully, and we have no hesitation in saying that it approaches nearer the ideal reference medical dictionary than any other which we ever saw. The definitions are accurate and complete, the pronunciation is the most approved and clearly indicated, while within the volume is a lot of useful matter not found in any other work of the sort. We cheerfully commend it to any one wishing an excellent modern dictionary.

---

LECTURES ON AUTO-INTOXICATION IN DISEASE. By Ch. Bouchard, Professor of Pathology and Therapeutics, Member of the Academy of Medicine and Physician to the Hospitals, Paris. Translated, with a Preface by Thomas Oliver, M.A., M.D., F.R.C.P., Professor of Physiology, University of Durham. Price, $1.75 net. The F. A. Davis Co., Publishers, 1914 and 1916 Cherry street, Philadelphia.

This is a series of lectures on the nature, forms and manner of self-poisoning of the individual, whether the poisons be introduced from without or generated within the body of man. Putrefactive processes and the development of poisonous alkaloids in the intestinal canal play an important part in many diseased conditions as at present understood. Such is the subject very ably discussed in this book, and the translator is correct in saying that none demands more serious study.

The lectures relate mainly to auto-intoxication in general, the toxic principles in urine and the part they play in producing uremia, the pathogenesis of uremia and the treatment of same, gastro-intestinal auto-intoxications, the pathogenesis of typhoid fever and the treatment of typhoid fever, auto-intoxication by bile, poisoning in diabetes, the cholera poison, etc. One chapter discusses the means of obtaining antisepsis and disinfection of the intestinal canal. These lectures are of great interest and value at the present day and it will fully repay any one to get them and read carefully.

GONORRHEA. Being a Translation of BLENNORRHEA OF THE SEXUAL ORGANS and its COMPLICATIONS. By Dr. Ernest Finger, Docent at the University of Vienna. William Wood & Co., New York.

The favorable reception which was accorded the two previous editions of this excellent work is the reason of the early appearance of this edition. No material change has been found necessary. although certain portions of this broad subject have elaborated in the light of recent investigations. Some additions are made, relating to the culture and staining of the gonococcus, and the diagnosis of posterior urethritis. The usual complications of the disease are fully discussed, such as balanitis, prostatitis, cystitis, etc. Gonorrhea in the female, and its complications, also receives proper and thorough consideration.

The volume contains thirty-six well-executed engravings and some full page plates in colors.

The book is well known as one of the best and most complete monographs on this subject, and the last edition cannot but perpetuate its popularity.

---

BIOGRAPHY OF EMINENT AMERICAN PHYSICIANS AND SURGEONS. Edited by R. French Stone, M.D., Indianapolis, Ind. Illustrated. Price, in cloth, $8.00. Carlon & Hollenbeck, Indianapolis.

Such information as is contained in this volume will often be found useful to every one. The editor presents here a short sketch of the lives and labors of the most eminent American physicians and surgeons, and his biography is one of the best that is now offered the profession. We note the absence of some names justly entitled to a place in a work of this sort, and indeed the presence of some others perhaps not so worthy. However, the selection of names and the space allotted each have been marked with good judgment generally, and the author has very wisely gone into some detail with the physicians and surgeons who have made American medicine famous, such as Rush, Physick, Dudley, Wistar, Agnew, Davis, Sims, Emmett, and others. The illustrations are generally good. We think any one will be well pleased with this book. It will be of especial value to those who are interested in the history of American medicine. Orders will be received by the editor, Dr. R. F. Stone, 16 W. Ohio street, Indianapolis.

---

INTERNATIONAL MEDICAL ANNUAL. A Work of Reference for Medical Practitioners, by Numerous Editors. Twelfth Year. 1894. Price, $2.75. E. B. Treat, Cooper Union, New York.

Every one is familiar with the Medical Annual. It is an annual digest of medical and surgical thought and progress, prepared by an able corps of editors. The present issue is an improvement and advance on its predecessors. More authors have contributed to its pages, and it contains more matter and illustrations. The general arrangement of material remains the same as in previous issues, and the price remains as heretofore. Among the illustrations are eight chromo lithographs and twenty-one full page half tone plates. The publishers have used every effort to enlarge the usefulness of the Annual, and we hope it will continue to receive the confidence of practitioners generally.

---

CLINICAL DIAGNOSIS. By Albert Abrams, M.D., Professor of Pathology, Cooper Medical College, San Francisco, Pathologist to the City and County Hospitals, etc. Third edition, revised and enlarged. Price, $2.75. E. B. Treat, Cooper Union, New York.

In revising this well known and practical work on diagnosis the author has added several useful synoptic tables, a chapter on insanity and a summary of the latest methods of diagnosis. In other respects the original character of the book has been preserved. The physical examination and diagnosis of diseases of the lungs and heart are completely described, and also the practical examination of the sputum; also the methods of examining the digestive system, and the diagnosis of the gastro-intestinal diseases. The chapter on the examination of the genito-urinary organs is one of the best and most practical. Other chapters relate in the same way to the nervous system, diseases of the skin, etc. We are sure this book will be found extremely useful and serviceable.

SAUNDERS' QUESTION COMPENDS. ESSENTIALS OF ANATOMY, INCLUDING THE ANATOMY OF THE VISCERA. By Charles B. Nancrede, M.D., Professor of Surgery and of Clinical Surgery in the University of Michigan. Fifth Edition. With an Appendix on the Osteology of the Human Body. 180 fine illustrations. W. B. Saunders, Philadelphia.

ESSENTIALS OF PHYSICS. By Fred. J. Brockaway, M.D., Assistant Demonstrator of Anatomy in the College of Physicians and Surgeons, New York. Second Edition, revised, with 155 illustrations. W. B. Saunders, Philadelphia.

ESSENTIALS OF NERVOUS DISEASES AND INSANITY, WITH SYMPTOMS AND TREATMENT. By John C. Shaw, M.D., Clinical Professor of Diseases of the Mind and Nervous System, Long Island College Hospital Medical School. Second Edition, revised, with 48 original illustrations. W. B. Saunders, Philadelphia.

ESSENTIALS OF PRACTICE OF PHARMACY. Second Edition, revised. By Lucius E. Sayre, Ph.G., Professor of Pharmacy and Materia Medica in the School of Pharmacy of the University of Kansas. W. B. Saunders, Philadelphia.

The four above named books are just out, and we think they are the best up to date compends on the market. They cover the subjects on which they are written in a clear and concise manner, and bring out many new points not commonly found in Question Compends. The work on Anatomy recommends itself, as it is based on the last edition of Gray's Anatomy. The Physics is very complete, giving as its name implies the *essentials* of Medical Physics. The Essentials of Nervous Diseases and Insanity is illustrated with cuts made from the original photographs. The descriptions of the various troubles that a general practitioner is most likely to meet with are good. The Essentials of Pharmacy is a well written book, and the arrangement of the drugs is very nice and convenient. We are also glad to see the Metric System compared with the old system, or Apothecaries' Weight. In this way American Pharmaceutical Students can see the advantage of the new over the old system. We recommend these books to all students and physicians who wish to get the most knowledge in the fewest words.

